# t(14;16)-positive multiple myeloma shows negativity for CD56 expression and unfavorable outcome even in the era of novel drugs

**DOI:** 10.1038/bcj.2015.6

**Published:** 2015-02-27

**Authors:** T Narita, A Inagaki, T Kobayashi, Y Kuroda, T Fukushima, M Nezu, S Fuchida, H Sakai, N Sekiguchi, I Sugiura, Y Maeda, H Takamatsu, N Tsukamoto, D Maruyama, Y Kubota, M Kojima, K Sunami, T Ono, M Ri, K Tobinai, S Iida

**Affiliations:** 1 Department of Hematology and Oncology, Nagoya City University Graduate School of Medical Sciences, Nagoya, Japan; 2 Department of Hematology and Oncology, Nagoya City West Medical Center, Nagoya, Japan; 3 Division of Hematology and Oncology, Department of Medicine, Kyoto Prefectural University of Medicine, Kyoto, Japan; 4 Department of Hematology and Oncology, Research Center for Radiation Casualty Medicine, Research Institute for Radiation Biology and Medicine, Hiroshima University, Hiroshima, Japan; 5 Department of Hematology and Immunology, Kanazawa Medical University, Uchinada, Japan; 6 Department of Hematology, National Cancer Center Hospital East, Kashiwa, Japan; 7 Department of Hematology, Kyoto Kuramaguchi Medical Center, Kyoto, Japan; 8 Division of Hematology and Oncology, Department of Internal Medicine, St Marianna University School of Medicine, Kanagawa, Japan; 9 Division of Hematology, National Hospital Organization Disaster Medical Center, Tokyo, Japan; 10 Division of Hematology and Oncology, Toyohashi Municipal Hospital, Toyohashi, Japan; 11 Department of Hematology and Oncology, Okayama University, Okayama, Japan; 12 Cellular Transplantation Biology, Kanazawa University Graduate School of Medical Sciences, Kanazawa, Japan; 13 Oncology Center, Gunma University Hospital, Maebashi, Japan; 14 Department of Hematology, National Cancer Center Hospital, Tokyo, Japan; 15 Division of Hematology, Respiratory Medicine and Oncology, Department of Internal Medicine, Faculty of Medicine, Saga University, Saga, Japan; 16 Division of Hematology/Oncology, Department of Internal medicine, Tokai University School of Medicine, Isahara, Japan; 17 Department of Hematology, National Hospital Organization Okayama Medical Center, Okayama, Japan; 18 Department of Internal Medicine III, Hamamatsu University School of Medicine, Hamamatsu, Japan

Multiple myeloma (MM) is an incurable plasma cell neoplasm developing through long-term multistep genetic events. Biological and clinical features of MM are associated with genetic aberrations such as chromosomal translocations involving the immunoglobulin heavy chain gene locus (*IGH*) and chromosomal hyperdiploidy involving odd number chromosomes. In particular, t(11;14)(q13;q32) involving the *CCND1* gene locus is characterized by lymphoplasmacytic morphology, frequent CD20 expression, an indolent clinical course, and a relatively favorable outcome in patients receiving high-dose therapy (HDT) with the aid of autologous stem cell transplantation (ASCT).^1^ In contrast, t(4;14)(p16.3;q32) involving *FGFR3/MMSET* gene loci is associated with concomitant possession of a chromosome 13q deletion, a common IgA subtype, and a relatively unfavorable outcome even in patients receiving HDT with ASCT. However, the overall prognosis of patients with MM harboring t(4;14) is improving since the introduction of proteasome inhibitors such as bortezomib.^2, 3^ Another important chromosomal aberration observed in approximately 5% of newly diagnosed MM is t(14;16)(q32;q23) involving the *c-musculoaponeurotic fibrosarcoma (c-MAF)* oncogene locus. Various studies have suggested that MM carrying t(14;16) is associated with less frequent extramedullary tumor formation and hypercalcemia and an unfavorable outcome. However, this remains controversial, as the number of patients analyzed in previous reports is relatively small.^4, 5, 6^ The aim of this study is to clarify the clinical features of patients with newly diagnosed MM (NDMM) harboring t(14;16) in Japan, especially focusing on phenotypic and karyotypic characteristics and treatment outcomes in the novel drugs era.

To clarify clinical and laboratory features and prognostic factors of t(14;16)-positive MM, a nationwide retrospective study was performed. Patients diagnosed as having symptomatic NDMM according to the International Myeloma Working Group (IMWG) criteria^7^ between 2002 and 2013 were enrolled after approval by each institutional ethical committee. The t(14;16) was detected by double color fluorescence *in situ* hybridization (FISH) using bone marrow samples. Expression of surface antigens such as CD56 and CD20 on MM cells was detected by flow cytometric analysis and defined as positive when more than 20% of the CD38-positive plasma cells were positive. Baseline characteristics at initial diagnosis, comorbidity, patient treatment regimens and clinical outcomes were collected using unified case report forms. Clinical responses were assessed according to criteria proposed by the IMWG.^8^ We also assessed 124 patients with NDMM without t(14;16) as a control, which was confirmed by global real-time quantitative reverse transcription-PCR-purified plasma cells and/or FISH analysis at the Nagoya City University Hospital.^9, 10^ The significance of differences in patients' demographics and clinical characteristics according to the status of t(14;16) were compared using the *χ*^2^ test (nominal variable) or the Mann–Whitney *U-*test (continuous variable). Overall survival (OS) was defined as the period between the date of initial diagnosis and the date of death. Progression-free survival (PFS) was defined as the period between the date of initial diagnosis and either the date of the first relapse or death of any causes. Survival curves were plotted by the Kaplan–Meier method and compared using log-rank and Breslow–Gehan–Wilcoxon tests. Data were analyzed with SPSS software (SPSS Inc., version 22, Chicago, IL, USA), and *P*<0.05 was considered statistically significant.

In total, 35 NDMM patients carrying t(14;16) were enrolled from 17 institutions. Clinical characteristics of the patients with or without t(14;16) are shown in Table 1. Median ages of the patients with or without t(14;16) at diagnosis were 64 and 69, respectively. Regarding the surface phenotypes of MM cells, none (0/23) of the t(14;16)-positive MM were positive for CD56 expression, whereas 79 of 111 (69%) t(14;16)-negative MM were CD56 positive(*P*<0.001). CD20 expression was more common in t(14;16)-positive MM (11/23, 48%) than in t(14;16)-negative MM (15/110, 14% *P*<0.001; Figure 1a). The proportion of patients with chromosomal aberrations determined by G-banded karyotyping was higher for patients with t(14;16) (16/30, 53%) than for those without (19/123, 15% *P*<0.001). Moreover, the patients with t(14;16) showed a higher frequency of the IgG subtype M protein (*P*<0.001), leukocytosis (*P*<0.001), thrombocytopenia (*P*<0.001) and hyperproteinemia (*P*=0.001), and a lower frequency of hypercalcemia (*P*=0.016), compared with those without t(14;16).

The OS of all patients with t(14;16) tended to be shorter than for those without t(14;16) (50% OS: 3.06 versus 4.40 years, *P*=0.113; Figure 1b), and a significant difference in OS was confirmed among patients who received one or more lines of treatment containing novel drugs such as bortezomib, thalidomide or lenalidomide (50% OS: 3.6 versus 5.4 years, *P*=0.013; Figure 1c). Poor performance status (PS⩾2), thrombocytopenia (<100 × 10^3^/μl) or high lactate dehydrogenase levels (>1.0 N) were significantly unfavorable prognostic factors for OS in patients with t(14;16)-positive MM (Figure 1d). On the other hand, advanced stage (International staging system stage III), anemia (<8.5 g/dl) and high β2-microglobulin level (⩾5.5 mg/l) were extracted as statistically significant unfavorable prognostic factors in t(14;16)-negative patients (Supplementary Figure 1). The PFS of patients with t(14;16) was also significantly shorter than for those without t(14;16) (50%PFS: 0.6 versus 1.2 years, *P*=0.007; Supplementary Figure 2a). In subgroup analysis, patients aged 65 years or younger and those who received ASCT also demonstrated shorter PFS when they carried t(14;16) (*P*=0.004 and *P*=0.031, respectively, Supplementary Figure 2b).

Our study must be interpreted carefully, because the institutions that enrolled the patients were not fully matched between t(14;16)-positive and -negative groups, indicating differences in treatment choices and supportive care systems. Despite this caveat, the first important finding regards the surface phenotype of MM cells. CD56 is generally expressed in 70–80%^11^ of patients with MM, as observed in 69% of the t(14;16)-negative cases in this study. In contrast, none of the t(14;16)-positive cases showed CD56 positivity. The underlying mechanism responsible for ectopic expression of CD56 in MM cells remains unknown. This difference is intriguing when considering biological behaviors of t(14;16)-positive MM cells, as CD56 is a neural cell adhesion molecule associated with cell-to-cell adhesion in the marrow microenvironment. Some recent reports have suggested inferior survival of CD56-negative compared with CD56-positive patients, although this remains controversial. Moreover, nearly half (48%) of the t(14;16)-positive MM cells expressed CD20. The CD20 antigen is frequently (42.9%) expressed in t(11;14)-carrying MM cells.^12^ Its expression in t(14;16)-carrying MM cells may represent their cellular origin from the immature plasma cell stage close to the lymphoplasmacytes. Second, chromosomal aberrations were detected in 53% of the t(14;16)-positive MM, suggesting high proliferative activity of the MM cells. On the other hand, the frequency of the abnormal G-banded karyotype found in NDMM patients is around 15–20% in Japan. Taken together, the data indicate that negativity for CD56 expression and high proliferative activity may predispose toward an unfavorable outcome of MM with t(14;16), even in the novel drugs era. The *c-MAF* oncogene encoding a basic leucine zipper transcription factor is transcriptionally activated as a result of t(14;16).^13^ The c-MAF oncoprotein upregulates transcription of *cyclin D2*, *integrin β7*, *CCR1*, *DEPTOR* and *Ark5,* all of which play crucial roles in malignant features of MM with t(14;16). Current therapeutic strategies are not satisfactory with respect to efficacy for MM with t(14;16), and unmet medical needs motivate ongoing searches for novel drugs targeting c-MAF itself or its downstream gene products to overcome its high-risk features.^14, 15^

## Figures and Tables

**Figure 1:**
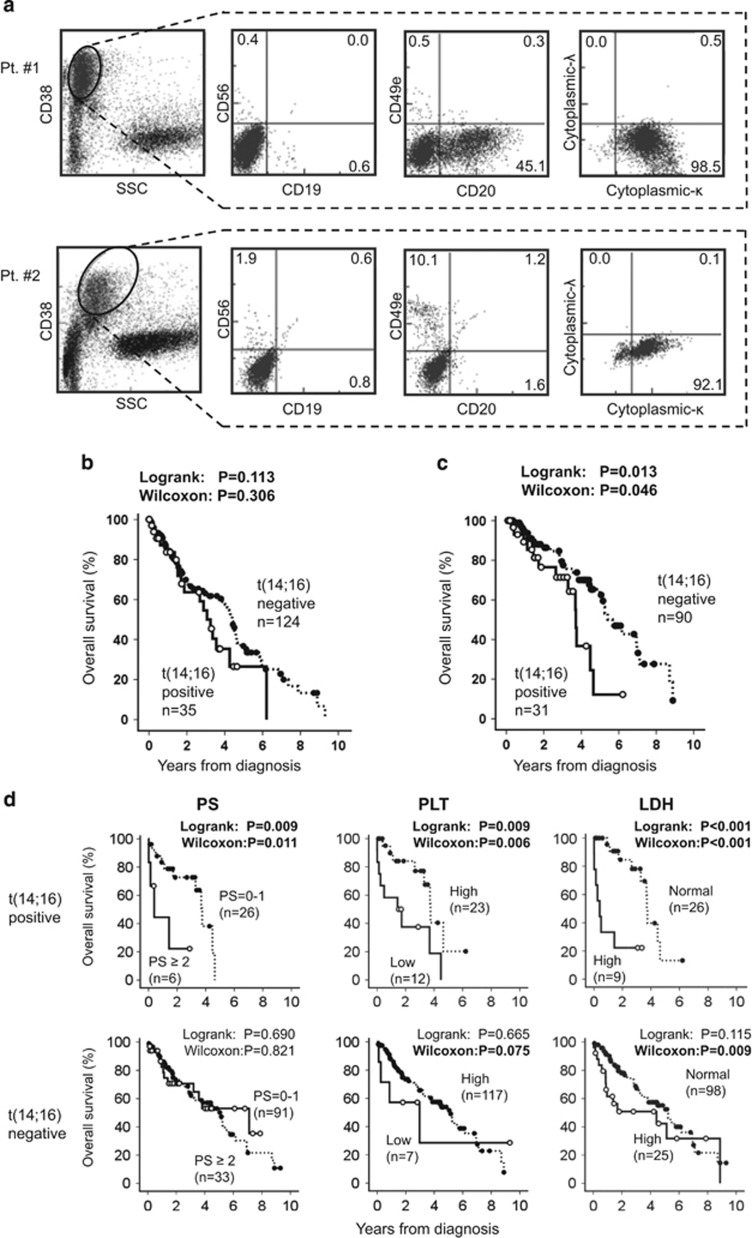
Flow cytometric analysis (FCM) of the representative t(14;16)-positive MM cells and overall survival (OS) of patients according to the presence or absence of t(14;16). (**a**) CD38^+^ plasma cells in bone marrow specimens obtained from patients with t(14;16) always showed negativity for CD56 expression (expressed lower than 20%) by FCM, as shown in Pt #1 and Pt #2. Moreover, CD20 is expressed more frequently in MM cells with t(14;16) than in those without t(14;16), as shown in Pt #1 (refer to Table 1). (**b**) OS curves for all MM patients according to the status of t(14;16) are plotted using the Kaplan–Meier's method. Censored cases are depicted by the dots. (**c**) OS curves of the patients who received one or more lines of novel drugs are plotted. (**d**) Statistically significant prognostic factors for the OS among t(14;16)-positive MM patients are shown with the corresponding survival curves based on performance status (PS), platelet count (PLT) and lactate dehydrogenase (LDH) values. They were also analyzed for patients without t(14;16) as shown below. The prognostification was determined by indexes of 0–1 or 2–4 for PS, higher (⩾100 × 10^3^/μl) or lower PLT (<100 × 10^3^/μl) and higher (>1.0 N) or normal serum LDH (⩽1.0 N).

**Table 1:** Patient demographics and their clinical characteristics

*Characteristics*	*t(14;16) Positive,* n*=35*	*t(14;16) Negative,* n*=124*	P *value*^a^
Age, years, median (range)	64 (36–86)	69 (34–95)	0.137
			
*Sex*			
Male	12/35 (34%)	53/124 (43%)	0.369
			
*ECOG PS*^b^			
2–4	6/32 (19%)	33/124 (27%)	0.360
			
*ISS*			
Stage	23/34 (68%)	53/119 (45%)	0.017
			
*M protein*			
IgG	27/35 (77%)	54/124 (44%)	<0.001^c^
IgA	2/35 (6%)	29/124 (23%)	
IgD	0/35 (0%)	7/124 (7%)	
Others^d^	6/35 (17%)	34/124 (26%)	
			
*Light chain*			
κ	17/35 (49%)	75/124 (60%)	0.208
			
*Bone lesion*			
Positive	23/35 (66%)	84/108 (78%)	0.153
			
*Upfront ASCT*			
Yes	8/35 (23%)	34/124 (27%)	
			
*Novel drugs*^e^			
Yes	31/35 (86%)	90/124 (73%)	
			
*Bone marrow laboratory results*			
*FISH*			
*c-MAF*	35/35 (100%)	−	
			
*G-band*			
t(14;16)	7/30 (23%)	−	
Abnormal^f^	16/30 (53%)	19/123 (15%)	<0.001
			
*CD20*			
Positive (⩾20%)	11/23 (48%)	15/110 (14%)	<0.001
			
*CD56*			
Positive (⩾20%)	0/23 (0%)	79/111 (71%)	<0.001
			
*Peripheral blood laboratory*			
*WBC*			
>10 000/μl	6/35 (17%)	1/124 (1%)	<0.001
			
*PB involvement*^g^			
Positive	10/35 (29%)	24/118 (20%)	0.304
			
*Hb*^h^			
<8.5 g/dl	15/35 (43%)	40/124 (32%)	0.244
			
*PLT*			
<100 × 10^3^/μl	12/35 (34%)	7/124 (6%)	<0.001
			
*cCa*^i^			
>11 mg/dl	2/35 (6%)	30/124 (24%)	0.016
			
*Total protein*			
⩾10.0 g/dl	18/35 (51%)	29/124 (23%)	0.001
			
*Albumin*			
<3.5 g/dl	16/19 (46%)	70/124 (56%)	0.260
			
*LDH*			
>1.0N^j^	9/35 (26%)	25/123 (20%)	0.494
			
*β2-microglobulin*			
⩾5.5 mg/l	21/34 (62%)	53/118 (45%)	0.083
			
*Creatinine*			
>2.0 mg/dl	30/35 (86%)	101/124 (81%)	0.559

Abbreviations: ASCT, autologous stem cell transplantation; c-MAF, *c-musculoaponeurotic fibrosarcoma;* ECOG, Eastern Cooperative Oncology Group; FISH, fluorescence *in situ* hybridization; ISS, international staging system; LDH, lactate dehydrogenase; PLT, platelet count; PS, performance status; WBC, white blood cells.

a *P* values were calculated using the *χ*^2^ test except CD56, WBC and cCa being calculated using the Fisher's exact test. Age was calculated using the Mann–Whitney *U-*test.

b PS proposed by ECOG.

c *P* value was calculated for IgG and non-IgG types.

d Including the IgM, IgD and BJP types.

e One or more lines of novel drugs; Bortezomib, Thalidomide and Lenalidomide.

f Genetic aberration without t(14;16).

g Peripheral blood involvement of myeloma cells.

h Hemoglobin.

i Compensation calcium value.

j 1.0 N means the upper limit of the normal range at each institution.

## Notes

SI received research funding from the Bristol-Myers Squibb Co., Chugai Pharmaceutical Co. Ltd., Taiho Phamaceutical Co. Ltd., Celgene K.K., Kyowa Hakko Kirin Co., Ltd., Ono Pharmaceutical Co. Ltd., Eli Lilly Japan K.K., Nippon Kayaku Co. Ltd. Honoraria from Janssen Pharmaceutical K.K., Celgene K.K. and Ono Pharmaceutical Co. Ltd. T.H.; Honoraria from Celgene Co., Ltd. KT received Honoraria from Celgene Co., Ltd. Research funding from Taiho Phamaceutical Co. Ltd., Celgene K.K., Ono Pharmaceutical Co. Ltd. Takeda Pharmaceutical Co. Ltd. M.R.; Research funding from Celgene Co., Ltd.
