# Supplementary material for: t(14;16)-positive multiple myeloma shows negativity for CD56 expression and unfavorable outcome even in the era of novel drugs
Source: Blood Cancer J. 2015 Feb 27;5(2):e285–. doi: 10.1038/bcj.2015.6 (PMC4349263; doi:10.1038/bcj.2015.6)
Supplement: Supplementary Information [file bcj20156x1.doc]

**SUPPLEMENTARY DATA**

**Figure S1. OS for patients with or without t(14;16) according to various clinical or laboratory factors.**

Results demonstrated that ISS, Hb, and β2-microglobin were valuable prognostic factors for t(14;16)-negative patients, but not for t(14;16)-positive patients. Abbreviations: L, Logrank; and W, Wilcoxon.

**Figure S2. Progression free survival (PFS).**

1. PFS curves for patients with or without t(14;16) were compared.
2. PFS according to various factors including patient age, treatment with or without high-dose therapy with the aid of autologous stem cell transplantation(ASCT), and response to the initial therapy were compared according to the status of t(14;16).
