# Supplementary figures and images for: t(14;16)-positive multiple myeloma shows negativity for CD56 expression and unfavorable outcome even in the era of novel drugs
Source: Blood Cancer J. 2015 Feb 27;5(2):e285–. doi: 10.1038/bcj.2015.6 (PMC4349263; doi:10.1038/bcj.2015.6)

Figure S1

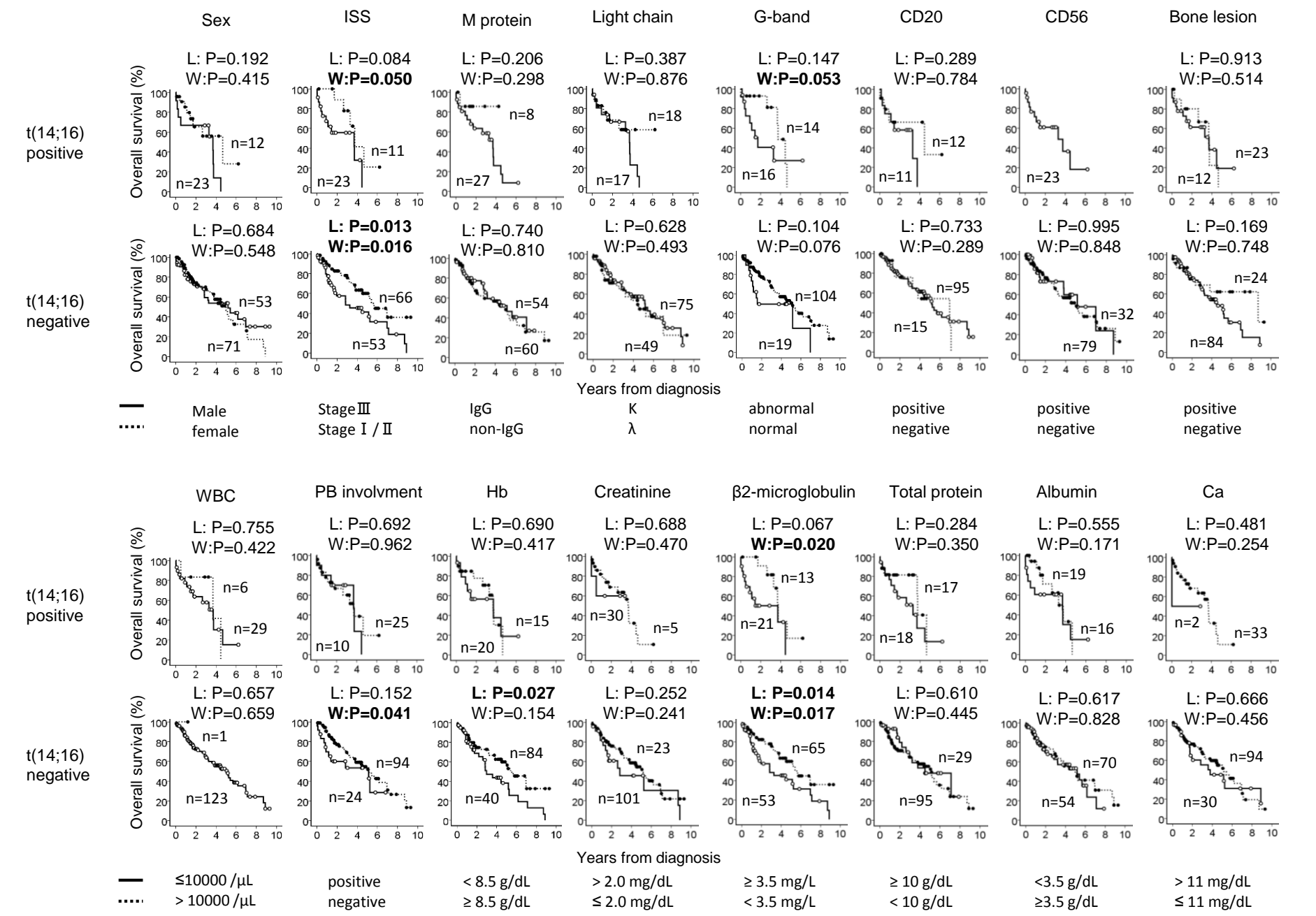

Supplement: Supplementary Figure S1 [file bcj20156x2.pdf]

Figure S2

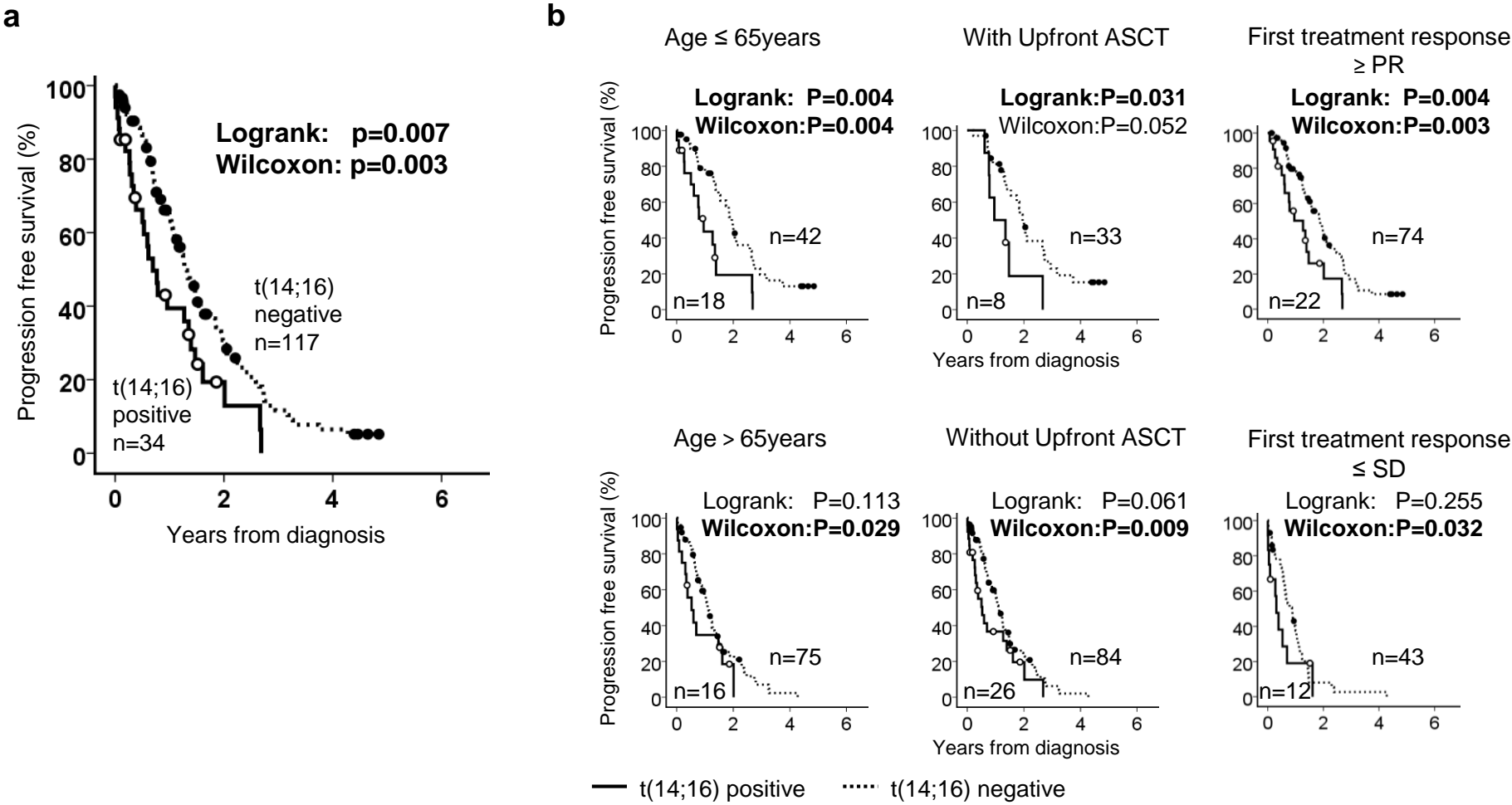

Supplement: Supplementary Figure S2 [file bcj20156x3.pdf]
